# Ionomics-metabolome association analysis as a new approach to the impact of dietary copper levels in suckling piglets model

**DOI:** 10.1038/s41598-023-28503-5

**Published:** 2023-01-20

**Authors:** Feng Zhang, Wen Yao, Xu Ji, Xiaodan Liu, Erhui Jin

**Affiliations:** 1 College of Animal Science, Anhui Science and Technology University, Chuzhou, 233100 China; 2 Anhui Province Key Laboratory of Animal Nutrition Regulation and Health, Chuzhou, 233100 China; 3 College of Animal Science and Technology, Nanjing Agricultural University, Nanjing, 210095 China; 4 Key Lab of Animal Physiology and Biochemistry, Ministry of Agriculture and Rural Affairs of the People’s Republic of China, Nanjing Agricultural University, Nanjing, 210095 China; 5 Anhui Province Key Laboratory of Livestock and Poultry Product Safety Engineering, Institute of Animal Science and Veterinary Medicine, Anhui Academy of Agricultural Sciences, Hefei, 230031 China; 6 Anhui AnFengT Animal Medicine Industry Co., LTD, Hefei, China

**Keywords:** Metabolomics, High-throughput screening, Animal physiology

## Abstract

Ionomics-metabolomics association analysis is a novel method to elucidating the potential mechanisms underlying the effects of dietary copper on the overall health parameters of suckling piglets model. Few studies have elucidated the relationship between the changes of ionic and metabolic homeostasis responses to dietary copper level. The growth performance data was obtained from 180 suckling piglets which access to different copper levels: 6 (low copper diet, LC), 20 (control diet, CON), and 300 (high copper diet, HC) mg·kg^−1^ copper (based on diet, supplementation from CuSO_4_), and offered ad libitum from d 14 until weaning at 40 d of age. Dietary high level copper (300 mg·kg^−1^) increased the ADG and ADFI during d 14 to 28 of piglets. Six elements (Mg, Na, K, P, Cu, and Mn) concentrations significantly changes in hair among the three treatment diets. The significant increased concentrations of Na and K, and decreased concentration of Mg and Mn in 300 mg·kg^−1^ than 20 mg·kg^−1^ copper diet was observed. In current study, with the increase in copper level from 20 to 300 mg·kg^−1^ in diet, the correlation between hair Na, K and Cu, Mn, Zn vanish. Hair Na and K were positively correlated with serum total antioxidant capacity (T-AOC) and negatively correlated with tumor necrosis factor-α (TNF-α). The hair Cu was negatively correlated with serum malondialdehyde (MDA), total bile acid (TBA). The fecal Cu was positively correlated with serum growth hormone (GH). The results suggested that the average daily gain (ADG) in 6 mg·kg^−1^ copper diet and the average daily feed intake (ADFI) in 20 mg·kg^−1^ copper diet were decreased than 300 mg·kg^−1^ copper diet during d 14 to 28 and the ADG was decreased in 6 and 20 mg·kg^−1^ copper diets in d 29 to 40 of piglets. Dietary 20 mg·kg^−1^ copper maintain ion homeostasis due to increase the number of positive correlations between macroelements-microelements in hair and serum. Significantly changed Na, K, Mg, Mn and Cu concentrations in hair can reflect the adverse effects of dietary 300 mg·kg^−1^ copper of suckling piglets. We believe our results may benefit people to gain a better understanding of the ion interactions and metabolic homeostasis of heavy metal elements that are critical to human and animal health.

## Introduction

Copper is a crucial trace element for the normal and healthy growth of mammals^1–5^. It has been found that at higher concentrations, copper exposure has been linked to liver disease, neurological disorders, reproductive abnormalities, and a multitude of other detrimental impacts on humans and animals^3,6^. The dose of copper that causes copper poisoning in monogastric animals must be at least 25 times more than its copper requirement, for pigs, this safe range can be expanded to 50 times, which means pigs can tolerate more than 250 mg·kg^−1^ copper in their diet. Once this threshold is exceeded, around 300 mg·kg^−1^ copper, it can accumulate in tissues and cause toxic damage, causing negative consequences such as poor feed consumption, decreased body weight, and blood-biochemical abnormalities contributes to abnormalities and diseases^3,7,8^.

Adding copper at 10 to 50 times the concentrations needed to meet requirements can substantially improve the rate of growth of swine and poultry and is a common practice^9^. During the last decades, copper has been used widely in antifungal agents, poultry feeds, and growth promoters^3,4,7^. Due to the pronounced growth promotion and antimicrobial activity of copper, it is widely used as a feed additive to control pig diseases and improve production performance, especially in weaning and fattening pigs with pharmacological copper (200–250 mg·kg^−1^)^2,3,10–14^. However, according to our previous studies, dietary high levels of copper are scarcely absorbed in the intestine and accumulated in intestinal chyme^8,15^, and 95% of the copper is expelled in animal feces^3^. The unreasonable addition of copper in the feed reverses the effect of promoting livestock growth into an inhibitory effect, even though it has a toxic effect^4^, an addition, which also presents serious environmental and public health concerns^3,4,7^. Our previous findings demonstrated that the copper contents was found to be significant accumulated in hindgut digesta with increased dietary copper levels in rats and piglets, the accumulation of copper alters the composition of intestinal microbiota in both animals^8,15^. More importantly, a significant correlation between the alterations of intestinal microbiota and serum TNF-α concentration was observed in rat experiments, which suggested that TNF-α could be the chief responder to microbiota shift under excessive copper exposure^1^. Otherwise, the correlation analysis of microbiome-metabolome suggested that dietary high-level copper alter the composition of the gut microbiota and modulate microbial metabolic pathways, which may further affect the health of suckling piglets^2^.

The advancement of ionomics technology has given a strong tool for the study of toxicological effects in recent years^3^. Ionomics is a novel multidisciplinary field that uses high-throughput elemental profiling technologies to investigate the mechanism underlying the cross-talk and trace element homeostasis in plants and animals^16^. Metabolomics, is the thorough profile of small-molecule metabolites in a live system, is increasingly being used to identify the involvement of numerous biomarkers in disease detection as well as insight into biological processes and toxicity mechanisms^3^. The rapid increase in omic data (such as metabolome, genome, and proteome) and recent advances in ionomics methods have facilitated the investigation of the dynamic relationship between trace elements and health or disease, which has been extensively investigated^16^.

A general procedure for ionomics-metabolome association analysis, (1) the biological sample of solid tissues, blood, and hair collection; (2) digestion and denaturation of collection samples; (3) measurement of elements by using high-throughput quantitative technologies, such as inductively coupled plasma mass spectrometry (ICP-MS), inductively coupled plasma optical emission spectrometry (ICP-OES), etc.; (4) after statistical analysis, the construction and changes of elemental correlation analyzed; (5) evaluate the association of ionomics profiles and differential metabolites, and construction of ionome-based models for classification or prediction of phenotypes of interest^16^.

Although, the integration of ionomics and metabolomics are scientifically significant for elucidating the impact of copper on animal health; however, related research has been scarcely reported. In the current study, the differential changes were identified of 13 elements in hair, blood, and feces obtained from suckling piglets, then the raw data on serum and fecal metabolism from our previous research were used to clarify the underlying relationship between ionomics profiles and piglet’s metabolism. We propose the following research hypothesis: copper supplementation in diet negatively influence the absorption and utilization of other elements, changes of the elemental composition in hair, serum, and feces related to serum biochemical markers and significant fecal metabolites, which in turn affects the health of suckling piglets. Through this work, the present study based on the ionomics-metabolome association analysis attempt to elucidate the impact of dietary copper on health parameters in the suckling piglets model.

## Results

### Growth performance

During phase 1 (d 14 to 28), compared with piglets fed the HC diet, the ADG in LC diet and the ADFI in CON diet were decreased (*P* < 0.05). With extension of feeding time (d 29 to 40), compared with the piglets that had HC diet, the ADG was decreased (*P* < 0.05) in LC and CON diets (Table 1).

**Table 1:** Effects of dietary copper on the growth performance of suckling piglets [Mean (SD)].

Item	Cu supplementation, mg·kg^−1^ diet			SEM	*P*-value
	LC	CON	HC		
Phase 1, d 14 to 28					
ADG, g	170.8 (30.1^b^)	183.3 (34.8^ab^)	197.5 (18.9^a^)	6.9	< 0.050
ADFI, g	49.8 (4.2^ab^)	45.5 (5.5^b^)	62.4 (15.2^a^)	2.8	< 0.050
Feed efficiency (G:F)	3.5 (0.8)	4.1 (1.1)	3.3 (0.8)	0.2	0.264
Phase 2, d 29 to 40					
ADG, g	203.0 (26.1^a^)	214.9 (16.3^a^)	163.7 (36.7^b^)	8.1	< 0.050
ADFI, g	120.1 (16.1)	117.9 (18.1)	118.2 (21.5)	4.1	0.985
Feed efficiency (G:F)	1.7 (0.4)	1.9 (0.3)	1.5 (0.5)	0.1	0.268
Overall, d 14 to 40					
ADG, g	185.9 (26.0)	198.0 (20.5)	183.4 (13.3)	2.8	0.443
ADFI, g	74.3 (4.6)	72.9 (9.0)	81.3 (13.5)	2.3	0.312
Feed efficiency (G:F)	2.5 (0.4)	2.8 (0.5)	2.3 (0.5)	0.1	0.279
Diarrhea rate, %	8.7 (4.0^a^)	6.6 (5.4^ab^)	2.2 (1.2^b^)	1.1	< 0.050

^a,b^Values within a row without a common superscript letter are significantly different (*P* < 0.05).

Compared with piglets fed the LC diet, the diarrhea rate in the HC diet was decreased (*P* < 0.05) during the overall period (Table 2).

**Table 2:** Effects of dietary copper on the diarrhea rate of suckling piglets [Mean (SD)].

Item	Cu supplementation, mg·kg^−1^ diet			SEM	*P*-value
	LC	CON	HC		
Diarrhea rate, %	8.7 (4.0^a^)	6.6 (5.4^ab^)	2.2 (1.2^b^)	1.1	< 0.050

^a,b^Values within a row without a common superscript letter are significantly different (*P* < 0.05).

### Ionomics profiles

**Figure 1:**
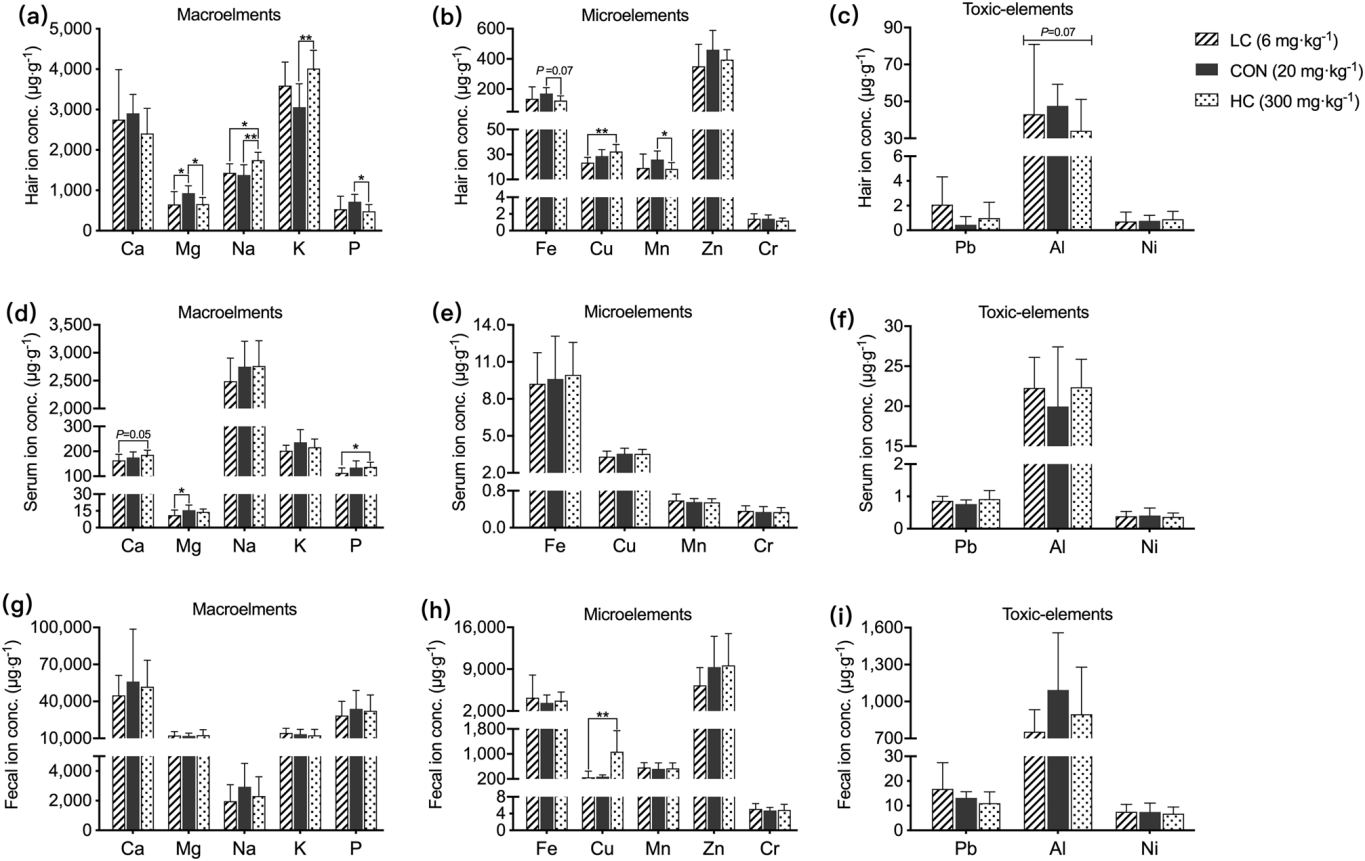
Effect of dietary copper levels on hair, serum and fecal ion concentrations in suckling piglets. Over all changes of 13 elements in hair (**a**,**b**,**c**), serum (**d**,**e**,**f**), and feces (**g**,**h**,**i**) among each dietary group. ^*^denote *P* < 0.05, ^**^denote *P* < 0.01.

Thirteen elements concentrations in hair, serum, and feces were described in Fig. 1. In hair, compared with piglets fed the CON diet, Mg, P, and Mn concentrations were decreased (*P* < 0.05) in HC diet, the Na, and K concentrations were increased (*P* < 0.01) in HC diet. Compared with piglets fed the LC diet, the Cu concentration was increased (*P* < 0.01) in HC diet (Fig. 1a–c). In serum, compared with piglets fed the LC diet, the Mg concentration was increased (*P* < 0.05) in CON diet, and the P concentration was increased (*P* < 0.05) in HC diet (Fig. 1d). No significant differences of Fe, Cu, Mn, Cr, Pb, Al, and Ni concentrations among each diet were observed (*P* > 0.05) (Fig. 1e,f). In feces, compared with piglets fed the LC diet, the Cu concentration was increased (*P* < 0.01) in HC diet (Fig. 1h). No significant differences of Ca, Mg, Na, K, P, Pb, Ai, and Ni concentrations among each diet were observed (*P* > 0.05) (Fig. 1g,i).

### Correlation between ionomics profiles and serum biochemical parameters

**Figure 2:**
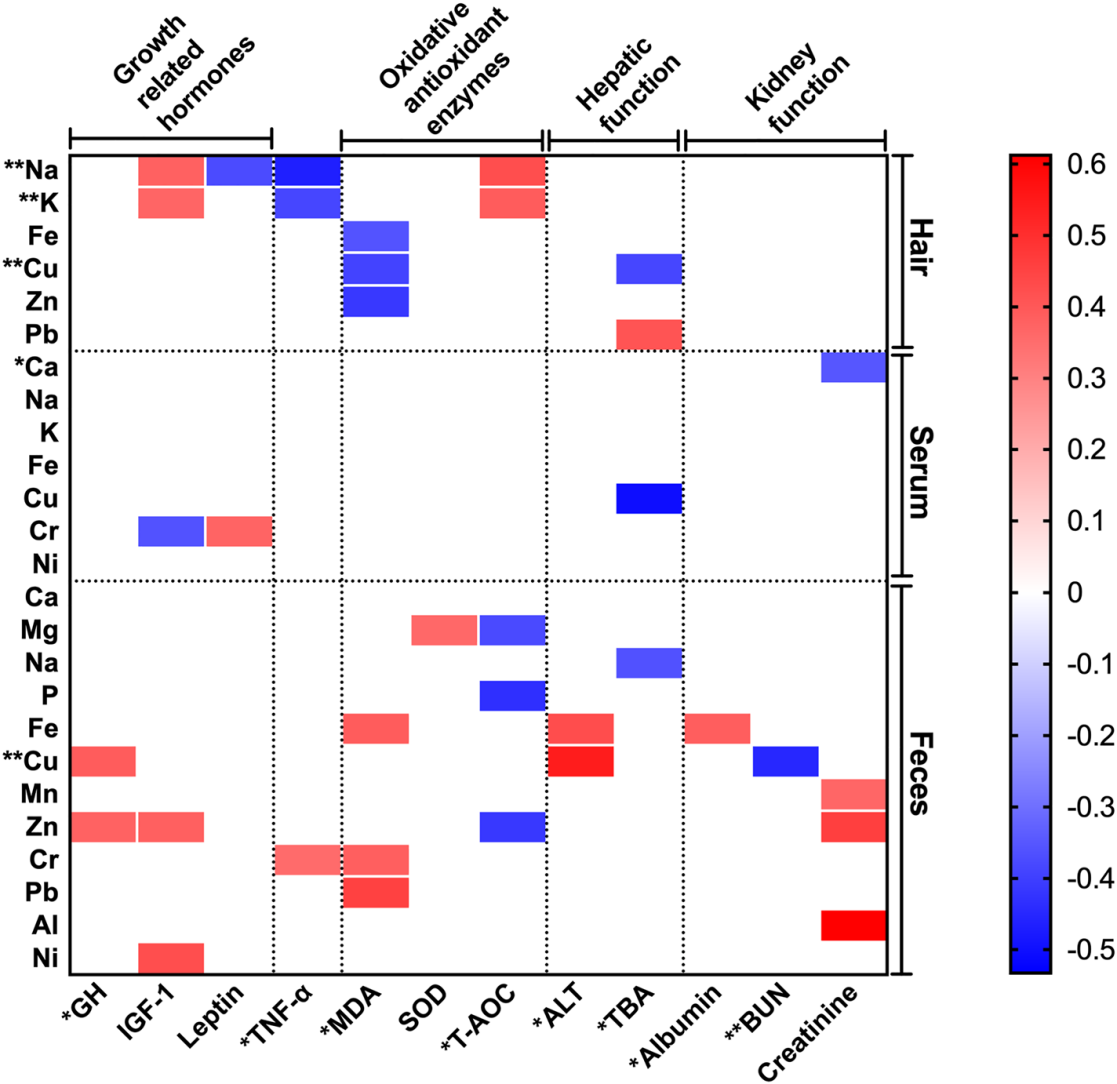
Correlation between ionomics profiles and serum biochemical parameters. The correlation which exited statistical significant were presented. The red represents a positive correlation (*P* < 0.05), the blue represents a negative correlation (*P* < 0.05), and the white shows that the correlation was not significant (*P* > 0.05). ^*^, ^**^significantly different among each copper diet, ^*^denote *P* < 0.05, ^**^denote *P* < 0.01.

The correlation between ionomics profiles and serum biochemical parameters were described in Fig. 2. Serum GH was positively (*P* < 0.05) correlated with fecal Cu and Zn; serum TNF-α was negatively (*P* < 0.05) correlated with hair Na and K and positively (*P* < 0.05) correlated with fecal Cr; serum MDA was negatively (*P* < 0.05) correlated with hair Fe, Cu, and Mn and positively (*P* < 0.05) correlated with fecal Fe, Cr and Pb; serum T-AOC was positively (*P* < 0.05) correlated with hair Na and K and negatively (*P* < 0.05) correlated with fecal Mg, P, and Zn; serum TBA was negatively (*P* < 0.05) correlated with hair and serum Cu and fecal Na and positively (*P* < 0.05) correlated with hair Pb; serum albumin was positively (*P* < 0.05) correlated with fecal Fe; serum blood urea nitrogen (BUN) was negatively (*P* < 0.05) correlated with fecal Cu.

### Correlation between ionomics profiles and fecal significant metabolites

**Figure 3:**
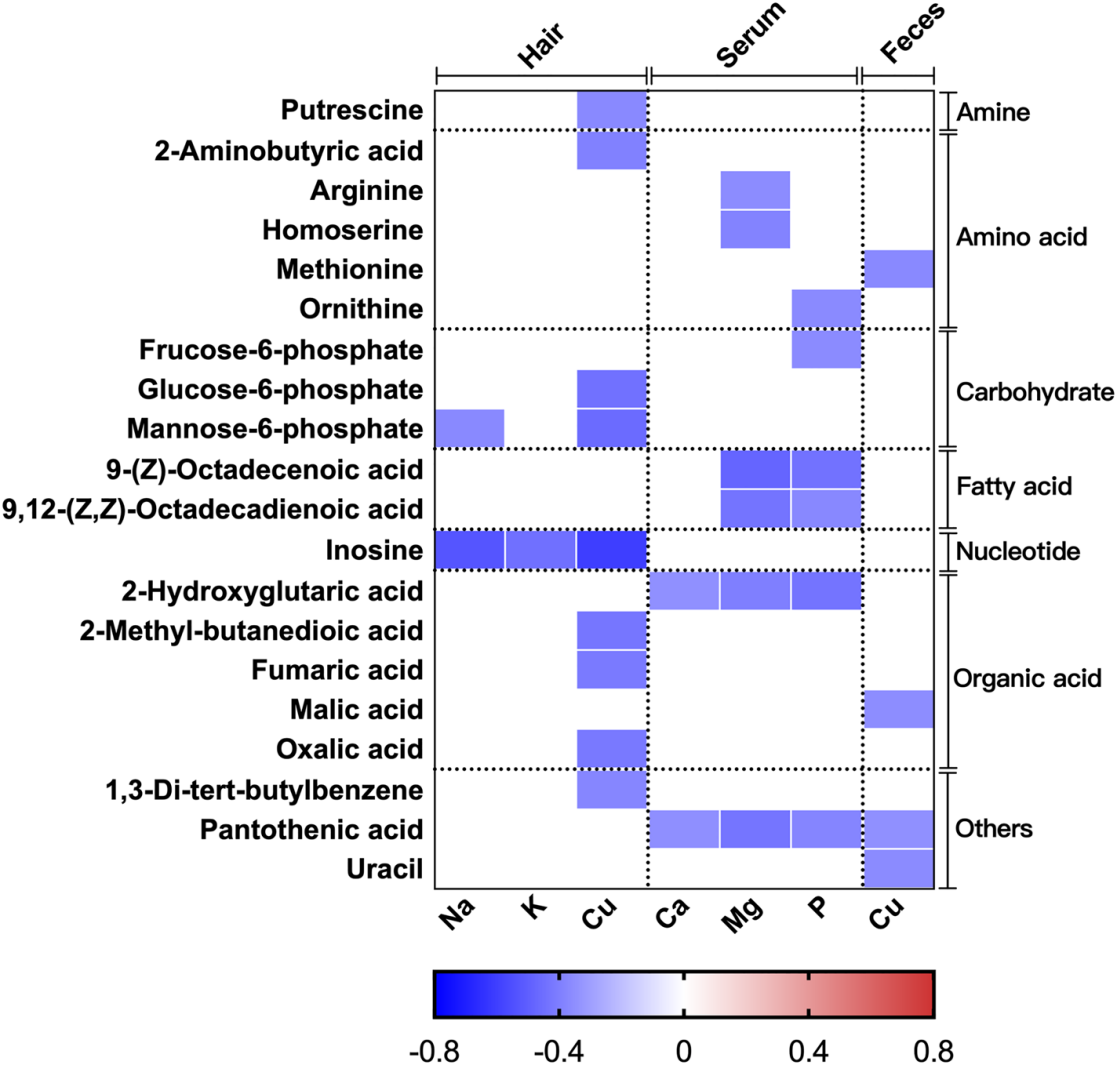
Correlation between ionomics profiles and fecal significant metabolites. The correlation which exited statistically significant were presented. The blue represents a negative correlation (*P* < 0.05), and the white shows that the correlation was not significant (*P* > 0.05).

The correlation between ionomics profiles and fecal significant metabolites were described in Fig. 3. Hair Na and K were negatively (*P* < 0.05) correlated with inosine; hair Cu was negatively (*P* < 0.05) correlated with putrescine, 2-aminobutyric acid, glucose-6-phosphate, mannose-6-phosphate, inosine, 2-methyl-butanedioic acid, fumaric acid, and oxalic acid; serum Ca, Mg, and P were negatively (*P* < 0.05) correlated with arginine, homoserine, ornithine, fructose-6-phosphate, 9-(Z)-Octadecenoic acid, 9,12-(Z,Z)-Octadecadienoic acid, 2-hydroxyglutaric acid, and pantothenic acid; fecal Cu was negatively (*P* < 0.05) correlated with methionine, malic acid, pantothenic acid, and uracil.

**Figure 4:**
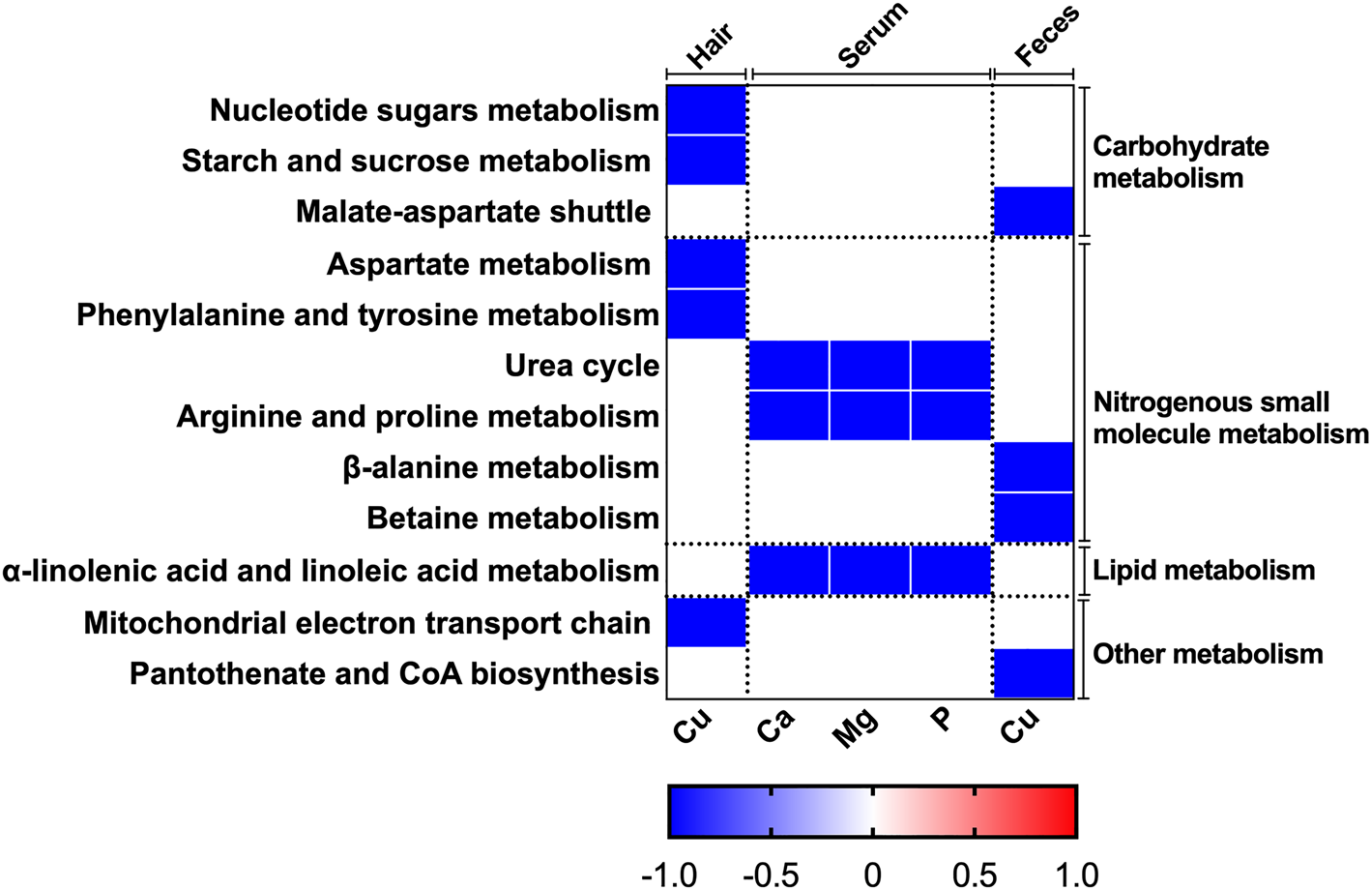
Correlation between ionomics profiles and metabolic pathways. The correlation which exited statistically significant were presented. The blue represents a negative correlation (*P* < 0.05), and the white shows that the correlation was not significant (*P* > 0.05).

The significant fecal metabolites (see Supplementary Table S2) which correlated with ionomics profiles were used for further metabolic pathways and metabolite set enrichment analysis (Fig. 4). The hair Cu was negatively correlated with nucleotide sugars metabolism, starch and sucrose metabolism, aspartate metabolism, phenylalanine and tyrosine metabolism, and mitochondrial electron transport chain pathways; serum Ca, Mg, and P were negatively correlated with urea cycle, arginine and proline metabolism, and α-linolenic acid and linoleic acid metabolism pathways; fecal Cu was negatively correlated with β-alanine metabolism, betaine metabolism, malate-aspartate shuttle, and pantothenate and coenzyme A (CoA) biosynthesis pathways.

## Discussion

This study explores a new approach of ionomics-metabolome association analysis to investigate the potential relationship between ionomics profiles in hair, serum and feces and differential metabolites in serum and feces. It has been previously found that a high Cu diet promoted the growth rate of pigs^13,17^. In our study, we observed that dietary 300 mg·kg^−1^ copper enhanced ADG and ADFI during d 14 to 28, but ADG and the G:F value decreased during d 29 to 40 of piglets, previous study showed that dietary 300 mg·kg^−1^ copper can increase serum GH concentrations in weaning pigs^18^, which suggesting that dietary high levels of copper can promote short-term growth. Several studies have revealed that the positive effects of high-grade copper in the feed of piglets are mainly growth promotion^19,20^ and antibacterial activity^13^, higher nutritional levels of Cu (as CuSO_4_) at levels of 100 to 250 mg·kg^−1^ improved growth performance in young pigs^13,17^. Results from our research seemed inconsistent with the results in the above research, and may have been due to the toxic effects of dietary 300 mg·kg^−1^ copper adversely influencing productive performance of suckling piglets. In our study, dietary 6 mg·kg^−1^ copper seems unable to meet the nutritional needs of suckling piglets when antibiotics are withdrawn from creep feed because the G:F value decreased compared with the 20 mg·kg^−1^ copper diet, and the diarrhea rate significantly increased compared with the 300 mg·kg^−1^ copper diet. The US National Research Council (NRC) has suggested dietary requirements for 5 to 25 kg nursery pigs and growing pigs of approximately 3 to 6 mg·kg^−1^ copper^13^. The determination of copper content was 5.9 mg·kg^−1^ in feed of our LC diet. Therefore, the reason for the G:F results in our study may be the increased diarrhea incidence, which in turn affects piglets’ weight gain in the 6 mg·kg^−1^ copper diet. In our study, when piglets were fed a 20 mg·kg^−1^ copper diet, they tended to gain more and eat less among the three diets across the overall experimental period, which would be adequate to meet the suckling piglets’ requirements.

Hair mineral analysis has become an interesting diagnostic tool in the assessment of health and nutritional status^21,22^. In our study, of the 13 elements tested, six elements (Mg, Na, K, P, Cu, and Mn) prominent concentrations changes occur between the three treatment diets. We observed the significant increased concentrations of Na and K, and decreased concentration of Mg and Mn in 300 mg·kg^−1^ than 20 mg·kg^−1^ copper diet. Previous studies examined hair trace element contents in women with type 2 diabetes (T2D), Alzheimer's disease (AD) patients, and prostate cancer (PC) patients, interestingly, the significantly elevated Na, K and decreased Mg, Mn were associated with T2D^23^, AD^24^ and PC^25^. Our results in hair were consistent with the above studies, which suggested that dietary 300 mg·kg^−1^ copper may increase the incidence of several diseases of suckling piglets. In our study, the hair Cu concentration significantly increased in 300 than 6 mg·kg^−1^ copper diet, which shows the same trend variations as dietary copper levels. The high level of hair Cu appeared to be associated with the risk of PC^16,25^, also suggested that dietary 300 mg·kg^−1^ copper may increase the risk of the development of diseases in piglets. As previously reported, human hair as an excretory system for trace metals, and demonstrated long-term stability of nutritional status^26^, and considering that concentrations of these elements are reported to be correlated with the diagnosis of several diseases^16,27^. These results revealed that the variation trend of Na, K, Mg, Mn and Cu concentrations in hair of suckling piglets can reflect the adverse effects of 300 mg·kg^−1^ copper diet on the health of piglets to some extent.

Compared with the significant changes in the concentrations of elements in hair, only three elements (Mg, P, and Cu) changed in serum and feces in our study. The results showed that the concentrations of most elements in serum were not directly affected by dietary copper. There have been many reports suggesting that imbalance of several elements in serum appeared to be one of the risk factors of many complex diseases^16^. To further understand the effects of dietary copper on the elemental homeostasis, we analyzed the correlations between each element (see supplementary results, Fig. S1). In the current study, the correlation between Na, K and Cu, Mn, Zn disappears in hair from 20 to 300 mg·kg^−1^ copper diet (Fig. S1b,c). These results indicated that the concentrations of Na and K, which increased in 300 mg·kg^−1^ copper diet affected its correlations with Cu, Mn, and Zn. As is well known, trace elements such as Cu, Zn, and Mn are essential for normal growth, disease resistance, production and reproduction in farm animals^28^. This also indicated that with extension of feeding time (d 29 to 40), instead of promoting growth, dietary 300 mg·kg^−1^ copper it is unfavorable to piglet growth. Combined with these findings, the adversely influence of dietary 300 mg·kg^−1^ copper on productive performance could be reflected by the correlations between hair Na, K and Cu, Mn, Zn of suckling piglets. Noteworthy changes were also observed in the correlations between macroelements and microelements or toxic elements with increased copper concentrations in the diet. In our study, the positive correlations were increased in 20 than 300 mg·kg^−1^ copper diet. This result showed the occurrence of altered interdependencies between the elements in serum, which reflects the change in elemental homeostasis. Previously, it was shown that the absorption, utilization, and excretion of many trace elements in animals are greatly affected by other trace elements^28,29^. These results suggest that competitive between high level copper and trace elements can result in trace element deficiencies, thus dietary 20 mg·kg^−1^ copper can maintain the elemental homeostasis in piglets due to maintenance of the interactions between elements.

To understand the relationship between the ion balance and suckling piglets’ health status, correlations between ionomics profiles and serum biochemical parameters were analyzed (Fig. 2). In our study, hair Na and K were positively correlated with T-AOC and negatively correlated with TNF-α. The results showed that there may be a potential link between hair Na and K concentrations and serum T-AOC and TNF-α concentrations. Our previous study indicated that TNF-α was decreased and T-AOC was increased in 300 than 20 mg·kg^−1^ copper diet^8^ (Supplementary Table S1). These results suggested that changes of Na and K concentrations in hair can reflect the effects of 300 mg·kg^−1^ copper diet on serum inflammatory response and antioxidant capacity of suckling piglets to some extent. The negative correlations between hair Cu with serum MDA and TBA were also observed in our study. The MDA content reflects the severity of body exposure to free radicals, which are crucial biomarkers in the oxidative stress process^30^. In our previous study, the serum MDA was decreased in 20 mg·kg^−1^ copper diet compared with 6 and 300 mg·kg^−1^ copper diet, suggesting that dietary 20 mg·kg^−1^ copper could effectively protect tissues from oxidative damage^8^ (Supplementary Table S1). From these results, it seems that there is a certain connection between increased hair Cu concentrations and oxidative stress induced by dietary 300 mg·kg^−1^ copper. In our study, fecal Cu concentration was positively correlated with serum GH, and the increased serum GH concentration in 300 mg·kg^−1^ copper diet was also observed (Supplementary Table S1). Consistent with the previous study that high copper concentrations (100 to 300 mg·kg^−1^) can increase the serum GH concentration of weaning pigs^18^. It was believed that the growth-promoting effect of copper was related to the GH axis, and might be generated by the stimulation of GH secretion^31^. It appears that dietary 300 mg·kg^−1^ copper increased the serum GH concentration and fecal Cu concentration, which further verified the correlation between fecal Cu and serum GH. The concentrations of Zn in hair and feces were positively correlated with serum MDA and T-AOC in current study. Zn has the function of stabilizing cell membrane structure and protecting free radicals from oxidative damage^32,33^, this confirmed the above correlations. Results suggested that Zn concentration could reflect the oxidative stress state of suckling piglets.

As we know, fecal metabolites reflect the final status of animal digestion, absorption, and metabolism of feed nutrients^8^. Our previous analysis of significant fecal metabolites showed that the capacity of dietary monosaccharide and protein absorption decreased, and the concentration of organic acids was increased in suckling piglets fed with 6 mg·kg^−1^ Cu diet (see supplementary Fig. S2), these suggest that 6 mg·kg^−1^ Cu supplementation harms the health of piglets when antibiotic withdrawn from the feed^8^. In this study, we are concerned about changes in the composition of elements in hair, which reflect the body’s metabolism changes^34^. The correlation and enrichment analysis showed that the Na and K in hair which significantly affected by dietary copper, were negatively correlated with fecal inosine (Fig. 4), consistent with the result that hair Na and K were negatively correlated with serum TNF-α (Fig. 3), due to the inosine is a purine metabolite and has a systemic anti-inflammatory effect^35^. The hair Cu was significantly increased in the 300 than 6 mg·kg^−1^ copper diet and negatively correlated with phenylalanine and tyrosine metabolism pathways (Fig. 4), dopamine β-hydroxylase (DBH) and phenylalanine hydrolase are two key enzymes in these pathways which its cofactor is copper^36,37^. The hair Cu was negatively correlated with the mitochondrial electron transport chain pathway (Fig. 4). Various enzymes in this pathway use copper as a cofactor, such as a cytochrome c oxidase and nicotinamide adenine dinucleotide (NADH) dehydrogenase^36–38^. The activity of the electron transport chain is related to the generation of reactive oxygen species (ROS) and the body's redox state^39,40^. In this study, dietary 300 mg·kg^−1^ Cu enhanced the mitochondrial electron transport chain pathway, which promoted the formation of ROS and affecting the redox status of piglets, these verified by the results of negative correlation between hair Cu and serum MDA (Fig. 2).

The fecal Cu content mainly comes from the accumulation of unabsorbed copper in the diet. In our study, fecal Cu was negatively correlated with the betaine metabolism pathway. Betaine is an important methyl donor which provides methyl groups is mainly catalyzed by betaine homocysteine transferase (BHMT), a cytoplasmic enzyme that relies on zinc activation^41^, due to the antagonism between copper and zinc^28^, suggested that high-level dietary copper hinders the absorption of zinc to a certain extent, and inhibits the activity of BHMT as well as the function of betaine methyl donor. In the process of betaine producing methionine, homocysteine is also a substrate for the enzyme action of BHMT. When the activity of BHMT decreases, the concentration of homocysteine in the blood rises, which has a certain relationship with vascular disease, thrombosis, and renal dysfunction^42,43^, these suggested that dietary 300 mg·kg^−1^ copper inhibited the methyl supply capacity of betaine and further affected protein biosynthesis of suckling piglets. These suggested that 300 mg·kg^−1^ copper did not support the methyl supply capacity of protein biosynthesis, which further devoid of integral health benefits for suckling piglets. Our previous study found that the concentration of pantothenic acid was decreased in the 300 mg·kg^−1^ copper diet^8^. In our study, serum Ca, Mg, P concentrations were negatively correlated with urea cycle. The urea cycle is the body's way of converting toxic ammonia into urea. The concentration of urea found in blood can assess how well the kidneys are functioning^44^. These results suggested that serum Ca, Mg, P concentrations may be associated with renal function of suckling piglets, which also verified the changes of serum BUN between 20 and 300 mg·kg^−1^ copper diet (supplementary Table S1).

## Conclusions

Our study suggested that a correlation exists between the changes in certain elements concentrations of hair, serum, and feces and inflammatory response, oxidative stress and antioxidant capacity of suckling piglets when fed with different copper levels in diets. In particular, significantly changed Na, K, Mg, Mn and Cu concentrations in hair reflect the adverse effects of dietary 300 mg·kg^−1^ copper of suckling piglets, and dietary 20 mg·kg^−1^ copper maintains the elemental homeostasis in piglets due to maintenance of the interactions between elements. Our results may benefit people to understand the heavy metal elements posing a critical concern to human and animal health from ion interactions and metabolic homeostasis perspective.

## Materials and methods

### Ethics statement

All animal experiments complied with the ARRIVE guidelines. This animal experimental protocol was implemented under the supervision of the Chinese Guidelines for Animal Welfare and Experimental Protocol and were approved by the Institutional Animal Care and Use Committee of Nanjing Agricultural University, China (NJAU-CAST-2015-098).

### Animal, housing, diets and sampling

The experiments were designed as described in our previous study^8^. A total of 180 piglets (1.11 ± 0.18 kg BW) from 18 multiparous Suhuai sows (second pregnancy) were sorted into blocks by their anticipated farrowing dates. Eighteen litters piglets were randomly assigned to three treatments (6 litters/treatment, 10 piglets per litter), each treatment included three replicates. The treatments (factors) included three levels of copper supplementation from Cu sulfate (CuSO_4_) according to our previous study^8^: (1) 6 mg·kg^−1^ copper (LC) diet, containing no supplemental copper; (2) 20 mg·kg^−1^ copper (CON) diet; or (3) 300 mg·kg^−1^ copper diet (HC). In each litter, all piglets were selected based on similar body weight (BW), BW and sex were balanced among the piglets, and all piglets were individually weighed within 72 h after farrowing. The piglets were trained to feed when 7 d old with a prefeeding period of 7 to 14 d, and the animal trials were conducted over 26 days (14–40 d), and the corn/soybean based diets were supplied throughout the experiment, meeting the nutritional requirements of the NRC^45^ (Table 3). The piglets were housed together with their sow during the experimental period, and with free access to creep feed and water, all piglets were hindered from having access and eating the sow feed^8^. Each litter was monitored three times a day, and creep feeders were refilled as needed. Wet creep feed was removed, dried, and weighed, and feeders were refilled approximately every 8 h. Creep feed consumption was recorded daily. Individual pigs were weighed at d 14, 28, and 40 d post farrowing. Piglet BW and creep feed consumption were used to calculate ADG and ADFI. The diarrhea rate of piglets was recorded daily and calculated as follows: Diarrhea rate (%) = the number of pigs with diarrhea × diarrhea days/(the total number of pigs × experiment days) × 100%, in which the “number of pigs with diarrhea” was defined as the number of piglets with diarrhea was observed each day^46^.

**Table 3:** Composition of the experimental diets [Mean (SD)].

Item	Cu supplementation, mg·kg^−1^ diet		
	LC	CON	HC
Ingredient (%)			
Corn	69.00		
Soybean meal	14.00		
Extruded soybeans	5.00		
Whey powder	5.00		
Fish meal	2.00		
Sucrose	2.00		
Soybean oil	1.00		
CaHPO_4_	0.60		
Calcium carbonate	0.40		
NaCl	0.20		
L-Lysine	0.30		
DL-Methionine	0.07		
L-Threonine	0.03		
Choline chloride	0.10		
Vitamin premix^a^	0.10		
Mineral premix^b^	0.20		
Measured nutritional level (%)^c^			
Dry matter	87.20		
Crude protein	17.90		
Crude fat	2.00		
Crude fiber	4.20		
Ash	4.80		
Measured Cu value, μg·g^−1^	5.90 (1.00)	23.40 (3.60)	295.30 (14.80)

^a^The vitamin premix provided per kilogram of diet: vitamin A, 10,000 IU; vitamin D_3_, 2500 IU; vitamin K_3_, 3.0 IU; vitamin B_5_, 40 mg; nicotinic acid, 60 mg; folic acid, 1 mg; biotin, 0.2 mg; vitamin B_6_, 4.0 mg; vitamin B_2_, 7.5 mg; vitamin B_1_, 5.0 mg; vitamin B_12_, 0.08 mg.

^b^The mineral premix provided per kilogram of diet: Zn (ZnSO_4_·H_2_O), 100 mg; Cu (CuSO_4_·5H_2_O), 125 mg; Mn (MnSO_4_·H_2_O), 60 mg; Fe (FeSO_4_·H_2_O), 120 mg; I [Ca(IO_3_)_2_], 0.6 mg; Se (Na_2_SeO_3_), 0.30 mg. NO addition of antibiotics.

^c^Measured values.

Three litters were chosen within each treatment at 40 d. Feces, blood, and hair samples were collected from four piglets/litter, selected based on average BW (half male and female). Hair samples of the head, back, and buttocks of the piglets were collected and mixed; Blood samples were collected from the anterior vena cava of piglets and stored in glass tubes with no anticoagulant and were allowed to clot at 4 °C before the serum harvest by centrifugation (15 min at 3, 500 g); serum, hair and fecal samples were stored at − 80 °C for subsequent analysis^15^.

### Ionomics analysis

Thirteen elements, including macroelements (Ca, Mg, Na, K, P), microelements (Fe, Cu, Mn, Zn, Cr), and toxic-elements (Pb, Al, Ni) were measured in hair, serum, and feces using inductively coupled plasma optical emission spectrometry (ICP-OES) (PerkinElmer, USA). Hair (200 μg), serum (1 mL), and dried feces (500 μg) were placed in a tube with 10 mL of a mixture of nitric acid (guaranteed reagent, GR) and perchloric acid (GR) (3:1 v/v). After digestion overnight, tubes were heated from 100 °C to 240 °C over approximately 3 h, and the resulting digests were brought to constant volume with double distilled-deionized water^15,47^. The standard liquid of Ca, Mg, Na, K, and P (1000 µg·mL^−1^) were mixed to prepare 5 mL mixed standards with 0.5 mol·L^−1^ HNO_3_. Take 0.5 mL mixed standard of Fe, Cu, Mn, Zn and Cr (1000 µg·mL^−1^), constant volume to 5 mL secondary mother liquor (100 µg·mL^−1^), then using secondary mother liquor prepare 10 mL standard with 0.5 mol·L^−1^ HNO_3_. Take 0.5 mL mixed standard of Pb, Al, and Ni (1000 µg·mL^−1^), constant volume 2 times and prepare 5 mL secondary mother liquor (10 µg·mL^−1^), then prepare 10 mL standard with 0.5 mol·L^−1^ HNO_3_. According to the instrument software settings, the determination of each standard of solution’s absorbance value, each sample determination of repeated three times, get the element of the standard curve, according to the standard curve of the conversion of each element contained in the samples.

### Serum biochemical parameters analysis and the correlation with ionomics profiles

Serum biochemical parameters were analyzed for GH, insulin-like growth factors-1 (IGF-1), leptin, and TNF-α using the enzyme-linked immunosorbent assay (ELISA) kits (Nanjing Jiancheng Bioengineering Institute, Nanjing, China), other indexes including MDA, superoxide dismutase (SOD), T-AOC, alanine aminotransferase (ALT), aspartate transaminase (AST), TBA, BUN, and creatinine content in serum was directly detected by using the commercial reagent kits (Nanjing Jiancheng Bioengineering Institute, Nanjing, China) with UV–VIS spectrophotometer (Thermo, USA) according to the manufacturer’s instructions. The analysis of serum biochemical parameters was done, and the results were published^8^, the details were shown in Supplementary Table S1, in which the raw data was used to analyze the correlation with ionomics profiles.

### Fecal metabolite profiles analysis

The fecal samples preparation for gas chromatography-mass spectrometer (GC-MS) analysis and data acquisition and processing were consistent with what we’ve already published (see supplementary materials and Methods)^8^. The fecal metabolites with variable important projection (VIP) value > 1.0 and one-way analysis of variance (ANOVA) *P* values < 0.05 were considered as significant metabolites among the three dietary groups^8^. The raw data of significant metabolites was used to analyze the correlation with ionomics profiles.

### Statistical procedures

Statistical analyses were performed using IBM SPSS Statistics (version 26) as in our previous study^8^. The Shapiro–Wilk test and Q–Q plots were used to assess if the assumption of data being normal distributed was met. If this assumption was not met, data were log transformed. For data found not to possess a normal distribution, the nonparametric Kruskal–Wallis test was utilized. Growth performance data and ionomics profiles were compared among the diets using a one-way ANOVA applying Tukey’s post hoc test. The correlation analysis was performed by the Pearson correlation test. Probability (P) values of < 0.05 indicated that the difference is statistically significant. GraphPad prism 9.0 software was used to visualize the data.

## Supplementary Information

Supplementary Information.

## Data Availability

Our data are available per request to the corresponding author.

## References

[1] Liao J (2022). Endoplasmic reticulum stress contributes to copper-induced pyroptosis via regulating the IRE1alpha-XBP1 pathway in pig jejunal epithelial cells. J. Agric. Food Chem..

[2] Yang P, Wang HK, Zhu M, Li LX, Ma YX (2021). Degradation kinetics of vitamins in premixes for pig: Effects of choline, high concentrations of copper and zinc and storage time. Anim. Biosci..

[3] Qiao N (2021). Metabolomics and transcriptomics indicated the molecular targets of copper to the pig kidney. Ecotoxicol. Environ. Saf..

[4] Li Y (2021). Long-term copper exposure promotes apoptosis and autophagy by inducing oxidative stress in pig testis. Environ. Sci. Pollut. Res. Int..

[5] Zhao G, Zhang T, Sun H, Liu JX (2020). Copper nanoparticles induce zebrafish intestinal defects via endoplasmic reticulum and oxidative stress. Metallomics.

[6] De Jong WH (2019). Toxicity of copper oxide and basic copper carbonate nanoparticles after short-term oral exposure in rats. Nanotoxicology.

[7] Huo H (2021). Copper exposure induces mitochondrial dynamic disorder and oxidative stress via mitochondrial unfolded protein response in pig fundic gland. Ecotoxicol. Environ. Saf..

[8] Zhang F, Zheng W, Xue Y, Yao W (2019). Suhuai suckling piglet hindgut microbiome-metabolome responses to different dietary copper levels. Appl. Microbiol. Biotechnol..

[9] Reece WO, Erickson HH, Goff JP, Uemura EE (2015). Dukes' Physiology of Domestic Animals Minerals.

[10] Zhou W (1994). The role of feed consumption and feed efficiency in copper-stimulated growth. J. Anim. Sci..

[11] Lu L (2010). Effect of dietary supplementation with copper sulfate or tribasic copper chloride on the growth performance, liver copper concentrations of broilers fed in floor pens, and stabilities of vitamin E and phytase in feeds. Biol. Trace Elem. Res..

[12] Ma YL (2015). Multitrial analysis of the effects of copper level and source on performance in nursery pigs. J. Anim. Sci..

[13] Villagomez-Estrada S (2020). Effects of copper and zinc sources and inclusion levels of copper on weanling pig performance and intestinal microbiota. J. Anim. Sci..

[14] Lin G (2020). Optimal dietary copper requirements and relative bioavailability for weanling pigs fed either copper proteinate or tribasic copper chloride. J. Anim. Sci. Biotechnol..

[15] Zhang F, Zheng W, Guo R, Yao W (2017). Effect of dietary copper level on the gut microbiota and its correlation with serum inflammatory cytokines in Sprague-Dawley rats. J. Microbiol..

[16] Zhang Y, Xu Y, Zheng L (2020). Disease ionomics: Understanding the role of ions in complex disease. Int. J. Mol. Sci..

[17] Ren P, Chen J, Wedekind K, Hancock D, Vazquez-Anon M (2020). Interactive effects of zinc and copper sources and phytase on growth performance, mineral digestibility, bone mineral concentrations, oxidative status, and gut morphology in nursery pigs. Transl. Anim. Sci..

[18] Wang J (2016). Influence of dietary copper on serum growth-related hormone levels and growth performance of weanling pigs. Biol. Trace Elem. Res..

[19] Fry RS (2012). Amount and source of dietary copper affects small intestine morphology, duodenal lipid peroxidation, hepatic oxidative stress, and mRNA expression of hepatic copper regulatory proteins in weanling pigs. J. Anim. Sci..

[20] Huang C (2015). Dietary sodium butyrate decreases postweaning diarrhea by modulating intestinal permeability and changing the bacterial communities in weaned piglets. J. Nutr..

[21] Li Y (2011). Trace element concentrations in hair of healthy Chinese centenarians. Sci. Total Environ..

[22] Apostoli P (2002). Elements in environmental and occupational medicine. J. Chromatogr. B Anal. Technol. Biomed. Life Sci..

[23] Skalnaya MG, Demidov VA (2007). Hair trace element contents in women with obesity and type 2 diabetes. J. Trace Elem. Med. Biol..

[24] Koseoglu E, Koseoglu R, Kendirci M, Saraymen R, Saraymen B (2017). Trace metal concentrations in hair and nails from Alzheimer's disease patients: Relations with clinical severity. J. Trace Elem. Med. Biol..

[25] Guo J (2007). Prediction of prostate cancer using hair trace element concentration and support vector machine method. Biol. Trace Elem. Res..

[26] Nakaona L, Maseka KK, Hamilton EM, Watts MJ (2020). Using human hair and nails as biomarkers to assess exposure of potentially harmful elements to populations living near mine waste dumps. Environ. Geochem. Health.

[27] Wang XO (1995). Multielement ICP-AES analysis of hair samples and a chemometrics study for cancer diagnosis. Microchem. J..

[28] Goff JP (2004). Dukes' Physiology of Domestic Animals Minerals.

[29] Patra RC, Swarup D, Sharma MC, Naresh R (2006). Trace mineral profile in blood and hair from cattle environmentally exposed to lead and cadmium around different industrial units. J. Vet. Med. A Physiol. Pathol. Clin. Med..

[30] Li R (2019). Different sources of copper effect on intestinal epithelial cell: Toxicity, oxidative stress, and metabolism. Metabolites.

[31] Yang W (2011). Effect of high dietary copper on somatostatin and growth hormone-releasing hormone levels in the hypothalami of growing pigs. Biol. Trace Elem. Res..

[32] Kolachi NF, Kazi TG, Afridi HI, Kazi NG, Khan S (2012). Investigation of essential trace and toxic elements in biological samples (blood, serum and scalp hair) of liver cirrhotic/cancer female patients before and after mineral supplementation. Clin. Nutr..

[33] Larson MR (2000). Social desirability and self-reported weight and height. Int. J. Obes. Relat. Metab. Disord..

[34] Kosanovic M, Jokanovic M (2011). Quantitative analysis of toxic and essential elements in human hair. Clinical validity of results. Environ. Monit. Assess..

[35] Ali-Sisto T (2016). Purine metabolism is dysregulated in patients with major depressive disorder. Psychoneuroendocrinology.

[36] Kim BE, Nevitt T, Thiele DJ (2008). Mechanisms for copper acquisition, distribution and regulation. Nat. Chem. Biol..

[37] Festa RA, Thiele DJ (2011). Copper: An essential metal in biology. Curr. Biol..

[38] Birsoy K (2015). An essential role of the mitochondrial electron transport chain in cell proliferation is to enable aspartate synthesis. Cell.

[39] Bell EL (2007). The Q(o) site of the mitochondrial complex III is required for the transduction of hypoxic signaling via reactive oxygen species production. J. Cell Biol..

[40] Di Lisa F, Ziegler M (2001). Pathophysiological relevance of mitochondria in NAD(+) metabolism. FEBS Lett..

[41] Teng YW, Mehedint MG, Garrow TA, Zeisel SH (2011). Deletion of betaine-homocysteine S-methyltransferase in mice perturbs choline and 1-carbon metabolism, resulting in fatty liver and hepatocellular carcinomas. J. Biol. Chem..

[42] Ganu R, Garrow T, Koutmos M, Rund L, Schook LB (2013). Splicing variants of the porcine betaine-homocysteine S-methyltransferase gene: Implications for mammalian metabolism. Gene.

[43] Pajares MA, Perez-Sala D (2006). Betaine homocysteine S-methyltransferase: Just a regulator of homocysteine metabolism?. Cell. Mol. Life Sci..

[44] Barmore W, Azad F, Stone WL, Barmore W, Azad F, Stone WL (2022). Physiology, Urea Cycle. StatPearls.

[45] NRC (2012). Nutrient Requirements of Swine.

[46] Yin L (2020). Effects of vitamin B6 on the growth performance, intestinal morphology, and gene expression in weaned piglets that are fed a low-protein diet. J. Anim. Sci..

[47] Lin Z-M (2014). Effects of nitrogen fertilization and genotype on rice grain macronutrients and micronutrients. Rice Sci..

