# Supplementary material for: Ionomics-metabolome association analysis as a new approach to the impact of dietary copper levels in suckling piglets model
Source: Sci Rep. 2023 Jan 20;13:1164. doi: 10.1038/s41598-023-28503-5 (PMC9859785; doi:10.1038/s41598-023-28503-5)
Supplement: Supplementary file 1 — Supplementary Information. [file 41598_2023_28503_MOESM1_ESM.docx]

**Ionomics-metabolome association analysis as a new approach to the impact of dietary copper levels in suckling piglets model**

Feng Zhang^1, 2,^ *, Wen Yao^3, 4^, Xu Ji^5^, Xiaodan Liu^1^, Erhui Jin^1, 2, 6^

^1^College of Animal Science, Anhui Science and Technology University, Chuzhou 233100, China;

^2^Anhui Province Key Laboratory of Animal Nutrition Regulation and Health, Chuzhou 233100, China;

^3^College of Animal Science and Technology, Nanjing Agricultural University, Nanjing, 210095, China;

^4^Key Lab of Animal Physiology and Biochemistry, Ministry of Agriculture and Rural Affairs of the People’s Republic of China, Nanjing Agricultural University, Nanjing, 210095, China;

^5^Anhui Province Key Laboratory of Livestock and Poultry Product Safety Engineering, Institute of Animal Science and Veterinary Medicine, Anhui Academy of Agricultural Sciences, Hefei 230031, China;

^6^Anhui AnFengT Animal Medicine Industry Co., LTD;

^*^Correspondence: zhangfeng@ahstu.edu.cn; Tel.: +86-19855519807

Journal name: Scientific Reports

**Supplementary materials and methods for fecal metabolite profiles analysis**

**Sample preparation for GC-MS analysis**

Fecal samples (100 mg) were transferred into 5 mL centrifuge tubes; 500 μL of ddH_2_O (4 °C) was added, and the tubes were vortexed for 60 s. Next, 1 mL of methanol (precooled at -20 °C) and 60 μL of heptadecanoic acid (0.2 mg·mL^-1^ stock in methanol), as an internal quantitative standard, were added and vortexed for 30 s. The tubes were then placed into an ultrasound machine at 25 °C for 10 min, incubated on ice for 30 min, and centrifuged for 10 min at 12,000 rpm (4 °C), after which 1.2 mL of the supernatant was transferred into a new centrifuge tube. Samples were blow-dried by vacuum concentration, and then 60 μL of 15 mg·mL^-1^ methoxyamine pyridine solution was added. The sample was vortexed for 30 s and reacted for 120 min at 37 °C. Finally, 60 μL of bis(trimethylsilyl)trifluoroacetamide (BSTFA) reagent (containing 1% trimethyl chlorosilane) were added into the mixture, reacted for 90 min at 37 °C, and centrifuged at 12,000 rpm 4 °C for 10 min, and the supernatant was transferred into a bottle for inspection; 20 µL of each sample extract were used for quality control (QC), and the remaining sample material was used for gas chromatography-mass spectrometer (GC-MS) test detection.

**GC-MS analyzed the fecal metabolites profiles**

The derivatized sample (1.0 µL) was injected in split mode into an Agilent 7890A (Agilent, CA, USA) system in a 20:1 split ratio using the autosampler. Gas chromatography was performed on an HP-5MS capillary column (Folsom, CA, USA) to separate the derivatives at a constant flow of 1 mL·min^-1^ helium. The injection temperature was 280 °C, the interface set to 150 °C, and the ion source adjusted to 230 °C. The temperature-rise programs were followed by an initial temperature of 60 °C for 2 min, 10 °C min^-1^ up to 300 °C, and a steady 300 °C for 5 min. Mass spectrometry (Agilent 5975C, Agilent, CA, USA) was determined in full-scan method with a 35 to 750 (*m·z*^-1^) range.

**GC-MS data acquisition and processing**

Following raw data collection, compounds were identified by comparing the mass spectra and retention indices of all detected compounds with their reference standards and database in the National Institute of Standards and Technology (NIST, https://www.nist.gov/srd) database and NEW Wiley 9 mass spectra library database ^1^. The SIMCA-p software (version 13.0, Umetrics, Umea, Sweden) was used to conduct multivariate statistical analysis. The acquired GC-MS data were processed with partial least squares-discriminant analysis (PLS-DA) ^1^. The metabolites with variable important projection (VIP) values > 1.0 and one-way analysis of variance (ANOVA) P values < 0.05 were considered as different metabolites among the three dietary groups. The MetaboAnalyst (v4.0, http://www. metaboanalyst.ca/faces/ModuleView.xhtml) online tool was used to process the metabolic pathways and metabolite set enrichment analysis ^2,3^.

**Supplementary results for the changes in** **correlation patterns among elements in hair, serum and feces**

Correlation patterns among elements at different dietary copper levels are presented in Fig. S1. In hair, the number of correlations in the macro-micro category (upper-left, Fig. S1a-c) in the CON group was more than other groups. Compared with the CON group, the relevance of Cu = ƒ(Mg), Mn = ƒ(Na, K), and Zn = ƒ(K) were lost in both the LC and HC groups. The relevance of Fe = ƒ(Mg), Cu = ƒ(Na, K), Mn = ƒ(Na, K), Zn = ƒ(Na, K), and Zn = ƒ(Ca, P) were lost in the HC group. In the toxic-micro and toxic-macro categories (upper-right and bottom-left, Fig. S1a-c), Ni = ƒ(Cr, Cu) and Al = ƒ(Na, K) were lost in both the LC and HC groups compared with the CON group, while Al = ƒ(Cr) and Pb = ƒ(-Cu, -Na) appeared in the HC group. In serum, in the macro-micro category (upper-left, Fig. S1d-f), Fe = ƒ(Ca, Mg, Na, P), Cu = ƒ(Ca), Mn = ƒ(Ca, Mg) and Cr = ƒ(P) were lost, while Cu = ƒ(Na, P) appeared in both the LC and HC groups. No significantly positive correlations were observed between toxic and micro or macro elements in the CON group. In feces, with dietary copper levels increased from 20 to 300 mg·kg^-1^, the negative correlations of Ca and P with most micro and toxic elements were lost, while the positive correlation of Mg and K were appeared (Fig. S1g-i).


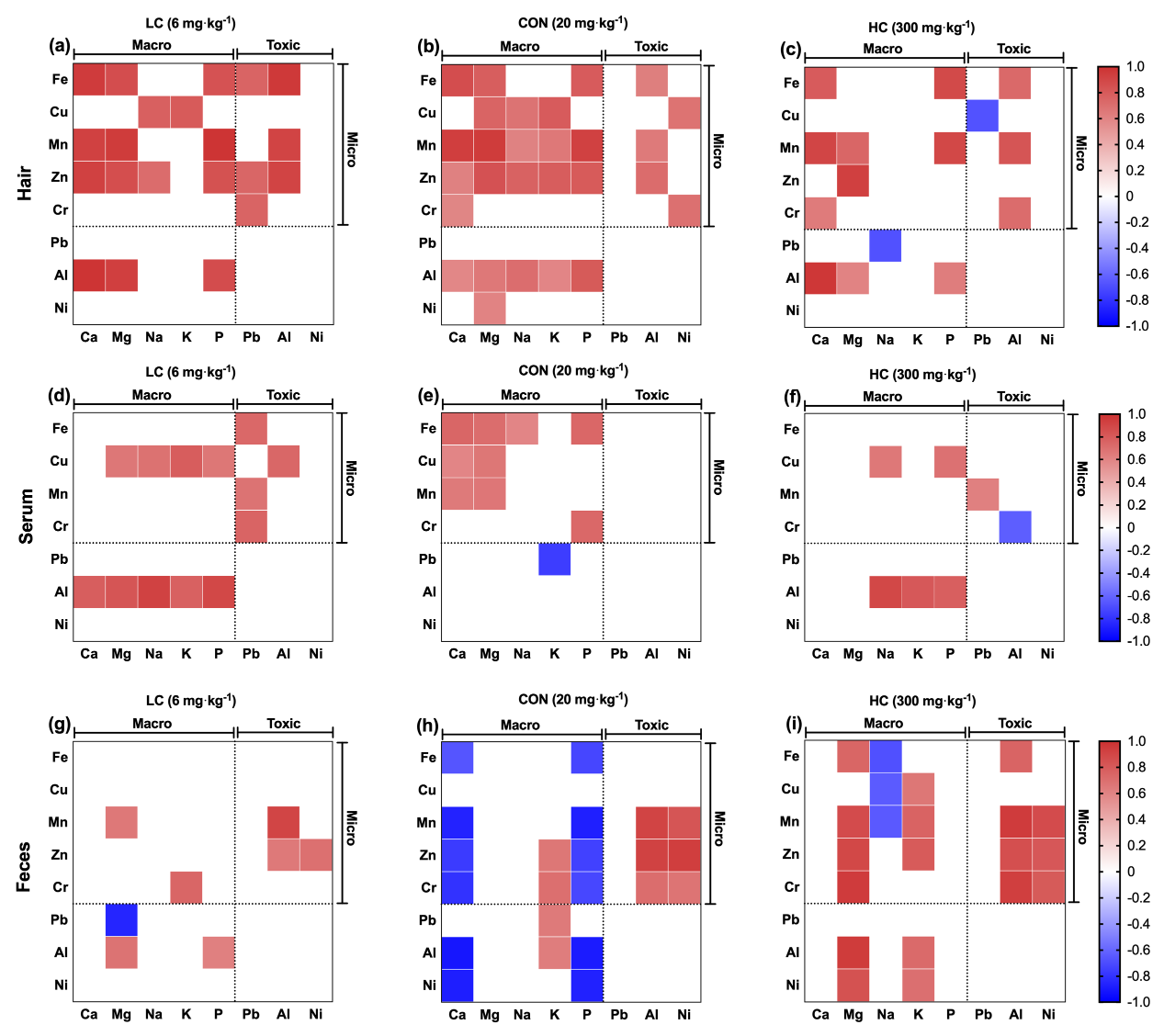


**Figure S1**. Changes in correlation pattern among hair, serum and fecal elements with different copper levels in diet. The macro-micro, macro-toxic and micro-toxic correlation pattern were presented with different dietary copper levels (6, 20 and 300 mg·kg^-1^) in hair (a, b and c), serum (d, e and f), and feces (g, h and i). The correlation which exited statistical significant were presented. The red represents a positive correlation (*P* < 0.05), the blue represents a negative correlation (*P* < 0.05), and the white shows that the correlation was not significant (*P* > 0.05)

**Supplementary tables**

**Table S1.** Effects of dietary copper on serum biochemical parameters of suckling piglets [Mean (SD)]^3^

| Item | Cu supplementation, mg·kg^-1^ diet | | | SEM | *P*-value |
| --- | --- | --- | --- | --- | --- |
|  | LC | CON | HC |  |  |
| Growth related hormones | | | | | |
| GH, ng·mL^-1^ | 1.2 (0.3^b^) | 1.2 (0.4^b^) | 1.7 (0.4^a^) | 0.1 | <0.05 |
| IGF-1, U·mL^-1^ | 9.5 (1.9) | 9.7 (2.2) | 11.0 (2.1) | 0.4 | 0.3 |
| Leptin, ng·mL^-1^ | 5.9 (0.7) | 5.8 (1.1) | 5.3 (1.6) | 0.2 | 0.5 |
| Inflammatory cytokine | | | | | |
| TNF-α, ng·L^-1^ | 70.4 (14.0^ab^) | 80.0 (24.8^a^) | 58.4 (17.8^b^) | 3.7 | <0.05 |
| Oxidative, antioxidant enzymes | | | | | |
| T-AOC, U·mL^-1^ | 2.1 (0.7^ab^) | 1.6 (0.4^b^) | 2.3 (0.8^a^) | 0.1 | <0.05 |
| MDA, nmol·mL^-1^ | 3.5 (0.8^a^) | 2.8 (0.5^b^) | 3.0 (0.5^ab^) | 0.1 | 0.1 |
| SOD, U·mL^-1^ | 142.9 (14.0) | 149.9 (7.5) | 138.5 (13.6) | 2.2 | 0.1 |
| Hepatic function | | | | | |
| ALT, U·L^-1^ | 11.5 (4.4^a^) | 7.6 (2.0^b^) | 8.6 (3.2^ab^) | 0.6 | <0.05 |
| AST, U·L^-1^ | 9.2 (2.3^a^) | 6.0 (1.7^b^) | 7.1 (2.1^ab^) | 0.4 | <0.01 |
| TBA, μmol·gprot^-1^ | 91.3 (43.1^a^) | 53.7 (24.9^b^) | 55.8 (18.6^b^) | 6.1 | <0.05 |
| Albumin, g·L^-1^ | 27.2 (4.2^a^) | 27.0 (3.4^a^) | 23.8 (2.2^b^) | 0.6 | <0.05 |
| Renal function | | | | | |
| BUN, mmol·L^-1^ | 4.0 (0.6^b^) | 4.6 (0.6^a^) | 3.7 (0.8^b^) | 0.1 | <0.01 |
| Creatinine, μmol·L^-1^ | 59.2 (13.0) | 70.4 (14.6) | 60.4 (15.2) | 2.6 | 0.1 |

^a,b^ Values within a row without a common superscript letter are significantly different (*P* < 0.05)

**Table S2.** Significant compounds in fecal samples among each group of nursing piglets

| Category | Metabolite | VIP^1^ | *P*-value |
| --- | --- | --- | --- |
| Carbohydrate | Arabinose | 1.41 | <0.01 |
|  | Glucose | 1.18 | <0.01 |
|  | Mannose-6-phosphate | 1.17 | <0.01 |
|  | Mannose | 1.16 | <0.01 |
|  | Frucose-6-phosphate | 1.15 | <0.01 |
|  | Glucose-6-phosphate | 1.14 | <0.01 |
| Amino acid | Lysine | 1.51 | <0.01 |
|  | Methionine | 1.40 | <0.01 |
|  | Alanine | 1.39 | <0.01 |
|  | Proline | 1.34 | <0.01 |
|  | Phenylalanine | 1.30 | <0.01 |
|  | Arginine | 1.29 | <0.01 |
|  | Valine | 1.28 | <0.01 |
|  | 4-Hydroxyproline | 1.24 | <0.01 |
|  | Leucine | 1.22 | <0.01 |
|  | Ornithine | 1.21 | <0.01 |
|  | Homoserine | 1.19 | <0.01 |
|  | 2-Aminobutyric acid | 1.19 | <0.01 |
|  | Isoleucine | 1.18 | <0.01 |
|  | Serine | 1.14 | <0.01 |
|  | Tyrosine | 1.08 | <0.01 |
|  | Aspartic acid | 1.07 | <0.05 |
|  | Threonine | 1.02 | <0.05 |
| Fatty acid | 9,12-(Z,Z)-Octadecadienoic acid | 1.31 | <0.01 |
|  | 9-(Z)-Octadecenoic acid | 1.19 | <0.01 |
| Amine | Putrescine | 1.36 | <0.01 |
|  | Ethanolamine | 1.16 | <0.01 |
| Polyol | Glycerol | 1.29 | <0.01 |
|  | Myoinositol | 1.14 | <0.01 |
| Organic acid | 2-Methyl-Butanedioic acid | 1.62 | <0.01 |
|  | Caffeic acid | 1.25 | <0.01 |
|  | Glycolic acid | 1.25 | <0.01 |
|  | Malic acid | 1.21 | <0.05 |
|  | Fumaric acid | 1.21 | <0.05 |
|  | 4-Hydroxybenzoic acid | 1.11 | <0.05 |
|  | Benzoic acid | 1.10 | <0.05 |
|  | Adipic acid | 1.06 | <0.05 |
|  | 2-Hydroxyglutaric acid | 1.06 | <0.05 |
|  | Oxalic acid | 1.03 | <0.05 |
|  | Lactic acid | 1.00 | <0.05 |
| Nucleotide | Inosine | 1.28 | <0.01 |
|  | Pseudouridine | 1.14 | <0.01 |
| Others | Pantothenic acid | 1.38 | <0.01 |
|  | 1,3-Di-tert-butylbenzene | 1.38 | <0.01 |
|  | Uracil | 1.33 | <0.01 |
|  | γ-Tocopherol | 1.26 | <0.01 |
|  | 3-Hydroxypyridine | 1.02 | <0.05 |

**Supplementary figure**


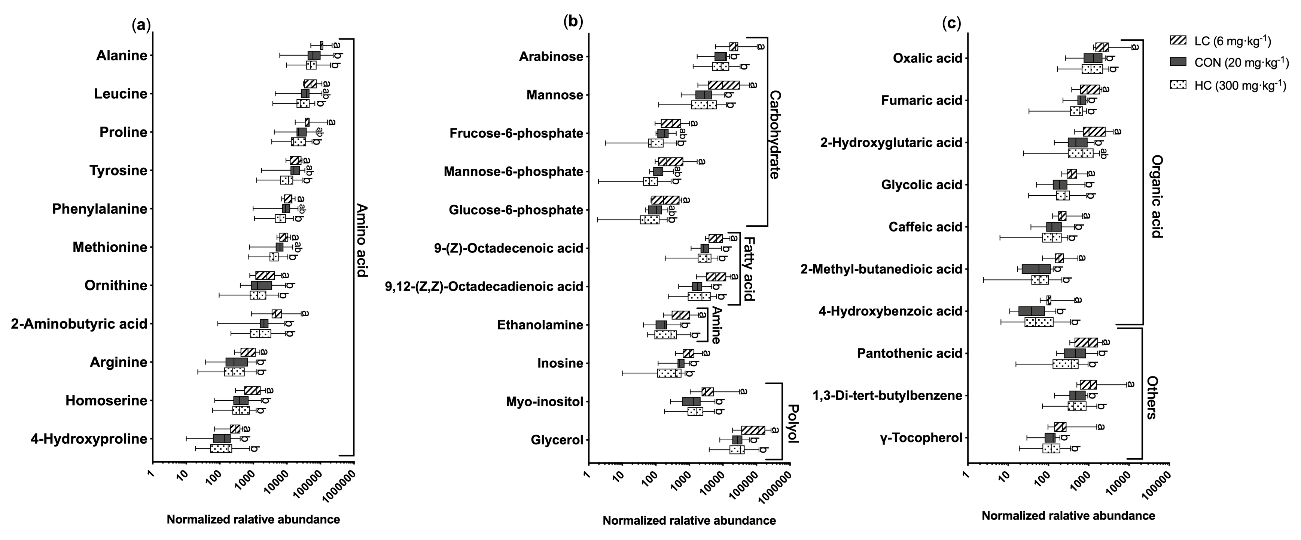


**Figure S2.** Effect of dietary copper levels on the normalized relative abundance of fecal significant metabolites. ^a, b^ denote *P* < 0.05

**References**

1 Sun, Y., Su, Y. & Zhu, W. Microbiome-metabolome responses in the cecum and colon of pig to a high resistant starch diet. *Front Microbiol* **7**, 779. <https://doi.org/10.3389/fmicb.2016.00779> (2016).

2 Xia, J., Psychogios, N., Young, N. & Wishart, D. S. MetaboAnalyst: a web server for metabolomic data analysis and interpretation. *Nucleic Acids Res* **37**, W652-660. <https://doi.org/10.1093/nar/gkp356> (2009).

3 Zhang, F., Zheng, W., Xue, Y. & Yao, W. Suhuai suckling piglet hindgut microbiome-metabolome responses to different dietary copper levels. *Appl Microbiol Biotechnol* **103**, 853-868. <https://doi.org/10.1007/s00253-018-9533-0> (2019).
